# Editorial: Vaccination of Special Populations: Protecting the Vulnerable

**DOI:** 10.3389/fimmu.2021.815550

**Published:** 2021-12-06

**Authors:** Daniel O’Connor, Viviana Moschese, Federico Martinon-Torres, Paolo Palma

**Affiliations:** ^1^ Oxford Vaccine Group, Department of Paediatrics, University of Oxford, Oxford, United Kingdom; ^2^ NIHR Oxford Biomedical Research Centre, Oxford University Hospitals NHS Foundation Trust, Oxford, United Kingdom; ^3^ Pediatric Immunopathology and Allergology Unit, Tor Vergata University Hospital, University of Rome “Tor Vergata”, Rome, Italy; ^4^ Genetics, Vaccines and Pediatric Infectious Diseases Research Group (GENVIP), Instituto de Investigación Sanitaria de Santiago and Universidad de Santiago de Compostela (USC), Galicia, Spain; ^5^ Translational Pediatrics and Infectious Diseases, Hospital Clínico Universitario and Universidad de Santiago de Compostela (USC), Galicia, Spain; ^6^ Centro de Investigación Biomédica en Red de Enfermedades Respiratorias (CIBER-ES), Madrid, Spain; ^7^ Clinical Immunology and Vaccinology Unit, Academic Department of Pediatrics, Ospedale Pediatrico Bambino Gesù-IRCCS, Rome, Italy; ^8^ Chair of Pediatrics, Department of Systems Medicine, University of Rome “Tor Vergata”, Rome, Italy

**Keywords:** vaccines, vulnerable populations, immunity, infectious diseases, immunisation

Despite the remarkable success of global vaccination programmes, vulnerable populations (VPs) — who are particularly susceptible to infectious diseases — are often undervaccinated and/or exhibit reduced vaccine immunogenicity. There are relatively limited immunogenicity and safety data of vaccines in VPs, including pregnant women, newborn and preterm infants, elderly, and individuals with chronic diseases. The purpose of this special issue was to gather the latest evidence about vaccine safety and immunogenicity in VPs, including epidemiology, basic immunology, systems vaccinology and human *in vitro* modelling data — required for optimal development and utilisation of vaccines for these special populations.

Primary immunodeficiency (PID) patients represent a population with profound susceptibility to infectious diseases. PID patients are also at high-risk of severe COVID-19. Although several COVID-19 vaccines are now available, inborn errors of immunity may lead to poor vaccine responses. Amodio et al., demonstrated most their PID patients developed both specific antibody and T cell responses — albeit humoral responses were of lower magnitude — following BNT162b2 SARS-CoV-2 vaccine, with no severe adverse events reported.

People leaving with HIV (PWH) represent another group of immune-compromised individuals with impaired vaccine responses. De Armas et al., carried out a detailed analysis of transcriptomic and immunological responses to 2-doses of pandemic influenza vaccine in HIV-infected children receiving anti-retroviral therapy (ART) to suppress viral load. The authors report a baseline molecular profile — of metabolic stress and immune activation — that was associated with low responders. Conversely, increased *CXCR5* expression — a homing marker expressed on T follicular helper (Tfh) — as well as an increased peripheral blood Tfh frequency and function following vaccination was seen in high responders.

Allogeneic haematopoietic cell transplantation (HCT) recipients have an altered vaccine-induced immunity and are highly susceptible to infectious disease. Therefore, immunisation of these patients against vaccine-preventable diseases is critical. Piekarska et al., assessed humoral immunity to hepatitis B virus (HBV) in HCT recipients, following recombinant hepatitis B surface antigen (rHBsAg) vaccine. Seroconversion was achieved in all HCT vaccinees, but severe chronic graft versus host disease (cGVHD) was associated with weak responses. Conversely, prior donor rHBsAg immunisation was associated with superior vaccine responses. Authors highlighted the benefit of both donor and recipient vaccination prior to HCT and propose an intensified vaccine schedule for weak responders.

Yellow fever (YF) vaccine is one of the most effective vaccines, evoking highly durable protection in healthy individuals. However, certain populations are at increased risk of rare but severe adverse events, leading to anxiety about the use of this live-attenuated vaccine in immune-compromised individuals. Valim et al., examined primary YF vaccine safety and immunogenicity in autoimmune disease (AID) patients with low disease activity and immunosuppression. Antibody levels were lower in autoimmune disease patients than healthy controls, but most individuals did seroconvert, and only mild adverse events were observed.

The immunological mechanisms underlying rotavirus (RV) vaccine protection are unclear. Gomez-Carballa et al., explore host transcriptome responses in children immunized with a live-attenuated RV vaccine (Rotateq) in comparison with children with rotavirus infection. This oral vaccine that replicates poorly in the gut evoked measurable changes in the blood transcriptome. This study showed similar molecular responses induced by vaccine and wild-type infection, including over-expression of genes associated with gastrointestinal disease and inflammation. However, machine-learning approaches were able to use the blood transcriptome to accurately distinguish vaccination and natural infection. This type of study has the potential to reveal the mechanisms of immune protection against rotavirus as well as enable a high-resolution assessment for vaccine safety and effectiveness.

Colucci et al., evaluated the immune and vaccine competence in children with steroid-sensitive nephrotic syndrome (SSNS). SSNS can lead to leakage of proteins into the urine and reduction in serum IgG levels. Moreover, immunosuppressive therapy used to treat these patients can strongly impact vaccine responses. Authors therefore evaluated the vaccine competence of SSNS patients by measuring vaccine-specific B cell responses, prior to administration of immunosuppressive treatment. Showing that while SSNS patients have reduced anti-tetanus and anti-HBV IgG levels, they had intact vaccine-specific B cell memory compared with controls.

Several reports have suggested certain vaccines may induce non-specific immunological effects that can modify susceptibility to unrelated infections. For example, childhood BCG and measles vaccines have been associated with a reduced risk in all-cause mortality i.e., beyond that expected due to tuberculosis (Tb) and measles. “Trained immunity” has been proposed as the mechanisms underlying these associations, which has been described as the modification of innate immune responses to induce “memory” of infection that results in enhanced and non-specific immune responsiveness to unrelated pathogens. Kleen et al., review BCG as a non-specific approach to modify immune responses to COVID-19 infection in at-risk populations. Evaluating whether in the setting of an emerging pandemic — before specific vaccines are available —the immune system could be modulated to improve immune responses. Authors propose to deploy the potential properties of BCG, a vaccine that has been in use for a century and with a well-defined safety profile, in the emerging outbreak setting.

While there is evidence that BCG reshapes innate immune responses to Tb-unrelated pathogens, the underlying mechanisms in early life are obscure. Angelidou et al., show distinct age-specific effects on newborn monocytes (cord blood) compared with adult monocytes, including distinct innate cytokine responses as well as “trained immunity”. Although they described greater TNF and IL-12p40 production in neonatal compared with adult monocytes following BCG stimulation, at day 7 BCG-trained adult monocytes demonstrated enhanced LPS-induced TNF production whereas newborn monocyte demonstrated tolerization. Moreover, BCG-trained newborn monocytes demonstrated an impaired BCG-induced production of lactate — a metabolite implicated in “trained immunity” in adults. These data showing age-associated differences in response to BCG may have important implications in the development of novel vaccines inducing non-specific protection.

Although the vaccine market is mostly for paediatric use, vaccine development is largely empirical and not tailored to meet the distinctions in innate and adaptive immune activation of early life. Beijnen and van Haren, evaluated vaccine-induced CD8^+^ T cell responses in children — reviewing age-specific molecular determinants that contribute to antigen cross-presentation. Where CD8^+^ T cells are desired, subunit-based vaccines need to be able to promote cross-presentation. There is evidence for adjuvant-induced cross-presentation, but little is known about whether and how adjuvants induce cross-presentation in early life. While young infants have higher frequency of CD8^+^ T lymphocytes, these have less diverse TCRs, reduced development of memory cells and have reduced cytotoxicity compared with adults CD8^+^ T cells. This review describes the sorting of soluble antigens for either classical MHC II presentation or cross-presentation on MHC class I, exploring critical steps in antigen cross-presentation and their competency in early life. Authors describe the inflammatory environment required to activate naïve CD8 T cells, and propose how adjuvant-induced cross-presentation can tailor distinct immune system early in life to induce potent CD8^+^ T cell responses.

Similar to other conditions associated with poorly regulated glucose metabolism, type 1 diabetes (T1D) confers increased risk to infection. There are clear genetic risk factors to T1D but the trigger is unclear, with some data suggesting that viral infection may induce development of T1D. These reports have stimulated significant debate about the use of live-attenuated viral vaccines in individuals at high-risk of T1D. Children with T1D are considered a special population and receive immunisation according to schedule recommendations for healthy subjects but with particular attention to pneumococcal and influenza vaccines. Here, Esposito et al. review the most effective and safe use of vaccines in individuals at risk of T1D or with overt T1D.

Routine childhood immunisation programmes have proven to be one of the most effective public health interventions. However, there are a number of barriers to vaccine uptake — including but not restricted to vaccine hesitancy. Olusanya et al., explored the barriers to childhood vaccine uptake and proposed recommendations for increasing vaccine compliance within the context of the COVID-19 pandemic. Authors advocate the use of more comprehensive evaluation of barriers to vaccination including social as well as individual determinants of health. Also, a multidisciplinary approach and antificial intelligence are proposed to promote optimal vaccine strategies.

Maternal immunisation offers protection to both pregnant women and their offspring, with transfer of vaccine-specific antibody to the infant reducing the incidence of neonatal tetanus and severe pertussis. Maternal immunisation has also been highlighted as strategy to prevent infant respiratory syncytial virus (RSV) and group B streptococcal (GBS) infections. Abu-Raya et al. are experts in infectious disease, vaccination, and maternal immunisation and detail a consensus paper summarizing the current literature on immunization during pregnancy and discuss current gaps related to vaccine safety and efficacy. Authors propose several priorities in future research to increase understanding of different aspects of maternal immunization to optimise protection for both the mother and child.

Qiu et al., report uptake of influenza and pertussis maternal vaccination, and factors associated with vaccine acceptance in high-income countries. Reasons for declining vaccine varied, but maternal safety concerns were a key factor associated with uptake. Knowledge gaps among pregnant women and lack of healthcare provider recommendation were important barriers for vaccine acceptance.

This Research Topic gathers some of the latest data about vaccine safety and immunogenicity in VPs, but also highlights the need for further work to improve vaccines and vaccine-uptake in these populations.

## Author Contributions

All authors listed have made a substantial, direct, and intellectual contribution to the work and approved it for publication.

## Conflict of Interest

FM-T reports his institution received trial fees from the GSK group of companies, Ablynx, Abott, Jansen, Medimmune, MSD Merck, Novartis, Novavax, Pfizer, Regeneron, Roche, Sanofi Pasteur and Seqirus for activities outside the presented work. FM-T also reports his institution received research grants from AstraZeneca, Jansen, MSD Merck and Pfizer for activities outside the presented work. FM-T also reports having received personal fees / honorarium from Biofabri, Novavax, Sanofi Pasteur, Seqirus, GSK group of companies, MSD Merck and Pfizer for activities outside the presented work.

The remaining authors declare that the research was conducted in the absence of any commercial or financial relationships that could be construed as a potential conflict of interest.

## Publisher’s Note

All claims expressed in this article are solely those of the authors and do not necessarily represent those of their affiliated organizations, or those of the publisher, the editors and the reviewers. Any product that may be evaluated in this article, or claim that may be made by its manufacturer, is not guaranteed or endorsed by the publisher.

